# Influence of Maternal Working Hours on Children’s Sleep: A Preliminary Study on Disparities Between Day and Night Shifts

**DOI:** 10.3390/clockssleep7040060

**Published:** 2025-10-23

**Authors:** Patrícia Andrade Nehme, Jefferson Santos, Ana Amélia Benedito-Silva, José Cipolla-Neto, Claudia R. C. Moreno

**Affiliations:** 1 Department of Health and Society, School of Public Health, University of São Paulo, Av. Dr Arnaldo, 715, São Paulo 01246-904, SP, Brazil; patricianehme07@gmail.com; 2 Department of Theory and Foundations of Education, Education Sector, Federal University of Paraná, Rua Rockefeller, 57-Rebouças, Curitiba 80230-130, PR, Brazil; jeffersosouza@ufpr.br; 3 School of Arts, Sciences and Humanities, University of São Paulo, Rua Arlindo Béttio, 1000-Ermelino Matarazzo, São Paulo 03828-000, SP, Brazil; aamelia@usp.br; 4 Department of Physiology and Biophysics, Institute of Biomedical Sciences, University of São Paulo, Av. Prof. Lineu Prestes, nº 1524, Butantã, São Paulo 05508-000, SP, Brazil; cipolla@icb.usp.br

**Keywords:** shiftwork, mothers and children, sleep, activity–rest

## Abstract

Background: Shift work necessitates alterations in daily routines, which can be detrimental to workers’ health and may also influence the activity and rest patterns of their children. Aim: The aim of this study was to evaluate the concordance between activity and rest parameters of mothers and their children, according to the mothers’ work shift (day vs. night). Methods: Twelve mother–child dyads participated in this study, including six mothers working night shifts and six working day shifts. All mothers followed a 12/36 h rotating schedule (07:00–19:00 for day shifts; 19:00–07:00 for night shifts). Participants wore actigraphy devices for 10 consecutive days. Sleep and motor activity parameters were analyzed using the Bland–Altman method. Results: Analysis of the five least active hours (L5) revealed increased nocturnal activity among the night shift group. The period of the 10 most active hours (M10) suggested greater activity in the day shift group, with a smaller difference between mother and child in the day shift group. The relative amplitude (RA) in the night shift group was lower among mothers compared to the day group. Interdaily stability (IS) was lower, and intradaily variability (IV) was higher in the night shift group, suggesting more irregular activity patterns. Bedtime data showed greater variability in the night shift group, with night shift mothers typically going to bed later than their children—a pattern that was also observed for wake times. In the day shift group, total sleep time did not differ between mothers and children; however, in the night shift group, discrepancies increased proportionally with total sleep duration. Sleep efficiency was lower among mothers in both groups, but the difference between mother and child was more pronounced in the night shift group. Conclusions: Night shift work among mothers appears to negatively affect both their own and their children’s activity and sleep parameters when compared to those in the day shift group.

## 1. Introduction

Sleep is a fundamental behavior for health, characterized by a complex state with numerous active processes, and it is essential for survival, given its restorative nature [1] (Roeths et al., 2000), with its importance becoming increasingly obvious. As a public health concern, it has also become a public healthcare issue, given that official data from different countries indicate that sleep disorders in contemporary society are increasingly prevalent [2] (Barone et al., 2023). Sleep impairment leads to long-term harm, being a predictor of diseases such as diabetes [3,4,5] (Engeda et al., 2013; Tan et al., 2018; Antza et al., 2021), arterial hypertension [6,7] (Li et al., 2021; Moon et al., 2021), and cardiovascular [8] (Miller et al., 2023) and metabolic diseases [9,10,11] (de Jonge et al., 2012; Broussard and Clain., 2022; Chaput et al., 2023), which lead to a worse quality of life and can contribute to premature death at all ages and social levels [12] (Cappuccio et al., 2017). Furthermore, lifestyle-related behaviors can also compromise sleep. Among these are a sedentary lifestyle [13,14] (Lechner et al., 2020; Ródenas-Munar et al., 2023), eating at irregular times [15,16,17] (Markwald et al., 2013; St-Onge et al., 2019; Papatriantafyllou et al., 2022), and the inversion of sleeping and waking times, usually due to unusual working hours, especially night work, which requires sleep during the day [18,19] (Giorgi et al., 2018; Visvalingam et al., 2019).

Shift work, including night work, is associated with insufficient sleep duration and, consequently, an increased risk of sleep disorders [20,21,22] (Kecklund and Axelsson, 2016; Ganesan et al., 2019; Kanki et al., 2023), leading to the desynchronization of biological rhythms with the environmental light–dark cycle. Additionally, there is emerging evidence that women who work overnight before and during pregnancy may have children with some type of health problem [23] (Nehme et al., 2019) in childhood and adolescence, such as overweight and obesity [24] (Strohmaier et al., 2018).

The detrimental effect of working irregular hours goes beyond biological aspects, as the family’s social context is also negatively affected [25,26] (Zhao et al., 2021; Tucker et al., 2021). These workers’ relationships with their spouses are harmed by atypical schedules [27] (Arlinghaus et al., 2019). There is evidence of the interaction between environmental factors, such as maternal employment, and negative health outcomes in children, including on sleep [28,29,30] (Kalil et al., 2014; Ananat and Gassman-Pines, 2021; Matsuoka et al., 2022), but such evidence is still insufficient [24,31] (Han et al., 2010; Strohmaier et al., 2018) to confirm the magnitude of such an impact. Studies that jointly analyze the social and health-related consequences of shift work are scarce [32,33] (Silva and Costa, 2023).

In this context, it is important to assess the extent to which maternal work schedules can affect workers’ sleep and how this impact can extend to their children’s sleep. This knowledge would allow us to verify whether the activity and sleep parameters of night workers are worse than those of day workers and whether there is agreement between the activity and sleep parameters of mothers and children. Our hypothesis is that maternal work hours change mothers’ activity and sleep parameters and, consequently, affect their children’s sleep quality. Thus, this study aimed to verify the agreement between activity/sleep parameters of mothers and children, according to the mothers’ work shift.

## 2. Results

The average working time in the professional category was 11.9 years (+8.4), differing between participants in the day shift (9.4 years + 9.2 years) and night shift (14.1 + 7.4 years). Regarding the time worked in the current shift, day shift workers had been working in this shift for an average of 9.6 years (+9.4 years) and night shift workers for 7.3 years (+7.5 years). None of the participants in either group were smokers.

Table 1 shows the sociodemographic data of the study participants and their children.

### Activity Parameters

**Figure 1:**
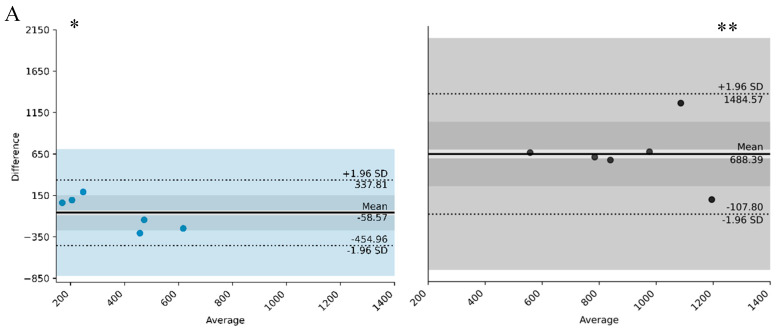

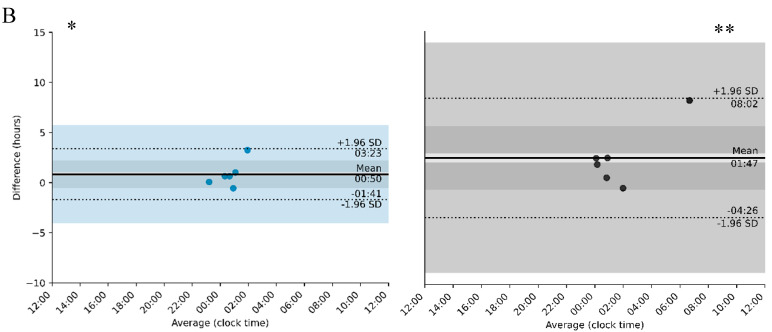
Period of 5 consecutive hours with less activity (average) (L5—(**A**) and L5 onset—(**B**)) of day workers and their children, compared to night workers and their children. * day shift and ** night shift. Solid lines represent the bias. Doted lines represent the concordance limit; Blue represent day group and grey represents the night group.

The results indicate a difference between day shift and night shift participants regarding the limits of agreement in the L5 parameter (Figure 1A), with a smaller difference between mother and child in the day shift group when compared to the night shift group. In other words, in the 5 h least active period, the children of women who worked during the day showed a similar pattern to their mothers. The average difference between mother and child in the day shift group was around −58.57 counts/min (mother < child), with agreement ranging between −454.96 counts/min and 337.81 counts/min. In the night shift group, the average difference was around 688.39 counts/min (mother > child), with limits of agreement between −107.8 counts/min and 1484.57 counts/min. On average, the L5 of a mother and child pair was between 500 counts/min and 1200 counts/min. The results indicate greater activity in the night shift group at night in L5, that is, during the 5 consecutive hours of lowest activity.

**Figure 2:**
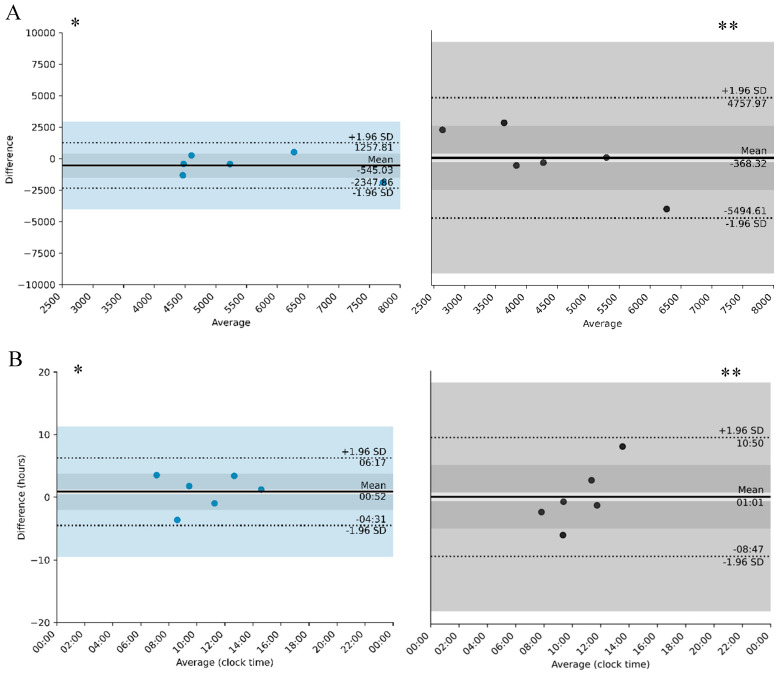
Period of 10 more active consecutive hours (average) (M10—(**A**) and M10 onset—(**B**)) for day workers and their children, compared to night workers and their children. * day shift and ** night shift. Solid lines represent the bias. Doted lines represent the concordance limit; Blue represent day group and grey represents the night group.

Figure 1A and Figure 2A refer to the differences in the magnitude of the activity of the variables L5 and M10; that is, they represent the average intensity of these variables.

Regarding the start time of the L5 period (Figure 1B), the average difference in bedtime between mother and child in the day shift group was 51 min (later for mother than for child), with agreement range between 1 h 42 min and 3 h 24 min. The start of L5 for the mother–child pair in the day shift group was between 11 p.m. and early dawn. In the night shift group, the average difference in bedtime was 1 h 47 (later for mother than for child), with a limit of agreement between −4 h 26 min and 8 h 18 min. The start time of L5 for a mother–child pair in the night shift group was between 11 p.m. and 7 a.m. In the night shift group, there is greater dispersion around the average of the differences between mother and child when compared to the day shift group.

Regarding M10 (Figure 2A), the average difference between mother and child in the day shift group was around 545.03 counts/min (mother > child), with agreement ranging between −2347.86 counts/min and 1257.81 counts/min. On average, the M10 of the mother and child pair was between 4500 counts/min and 7500 counts/min. In the night shift group, the mean difference was −368.32 counts/min (mother < child), with limits of agreement between −5494.61 counts/min and 4757.97 counts/min. On average, the M10 of the mother and child pair was between 2500 counts/min and 6500 counts/min. Greater activity is noted in the day shift group. A difference was observed between day shift and night shift workers regarding the limits of agreement in the M10 parameter, suggesting a smaller difference between mother and child in the day shift group when compared to the night shift group.

There is little difference between mother and child in the day shift group when compared to the night shift group regarding the limits of agreement in the start time of M10 (Figure 2B). The average difference between mother and child in the day shift group was 53 min, with agreement ranging between −4 h 22 and 6 h 17. On average, the start of M10 for the mother and child pair was between 7:00 a.m. and 2:30 p.m. In the night shift group, the average difference was 1 h 02, with limits of agreement between −8 h 47 and 10 h 51. The start time of M10 for the mother and child pair was between 7:00 a.m. and 2:00 p.m. The difference between mother and child increases for later onsets of M10.

The limits of agreement in the IS (interdaily stability) parameter suggest that the difference between mother and child is similar between day shift and night shift workers. The mean difference between mother and child in the day shift group was −0.01, with agreement ranging between −0.30 and 0.28. On average, the IS of a mother and child pair was between 0.50 and 0.65. In the night shift group, the average difference was around 0.03 (mother > child), with limits of agreement between −0.20 and 0.27. The IS of a mother–child pair was between 0.20 and 0.40, suggesting less stability between days when compared to the day shift group.

The intraday variability (IV) parameter presents little difference between day shift and night shift workers regarding the limits of agreement between mother and child. The mean difference between mother and child in the day shift group was 0.04, with agreement ranging between −0.19 and 0.2. On average, the IV of a mother and child pair was between 0.6 and 0.9. In the night shift group, the mean difference was 0.15, with limits of agreement between −0.36 and 0.67. The intraday variability of a mother–child pair was between 0.5 and 1.1, suggesting greater intraday variability when compared to the day shift group.

Regarding relative amplitude (RA), both groups show marked differences. In the night shift group, the relative amplitude of all mothers was smaller than that of their children. The mean difference between mother and child in the day shift group was 0.01, with agreement ranging between −0.13 and 0.16. On average, the RA of a mother and child pair was between 0.7 and 0.9. In the night shift group, the mean difference was −0.22, with limits of agreement between −0.41 and −0.04. This parameter for a night shift mother–child pair was between 0.5 and 0.7, suggesting a smaller difference between activity and rest when compared to the day shift group.

The limits of agreement in bedtime suggest little difference between mother and child in the day shift group when compared to the night shift group. The average difference between mother and child in the day shift group was 8 min (mother sleeps later than child), with agreement ranging between −2 h 14 and 2 h 17. On average, a mother and child’s bedtime was between 11:00 p.m. and early dawn. In the night shift group, the average difference was 2 h 17 (mother sleeps later than child), with a limit of agreement between −11 h 40 and 15 h 37. A mother and child’s bedtime started between 11:00 p.m. and 8:00 a.m. The night shift group shows greater dispersion around the mean difference compared to the day shift group.

There is little difference between mother and child in the day shift group when compared to the night shift group regarding the limits of agreement in wake time. The average difference between mother and child in the day shift group was −40 min (mother wakes up before child), with agreement ranging between −4 h 37 and 3 h 18. On average, a mother and son pair’s wake time was between 7 a.m. and 10 a.m. In the night shift group, the average difference was 1h 4, with limits of agreement between −9 h 45 and 11 h 53. On average, a mother and child pair’s wake time was between 7 a.m. and 3 p.m. The difference between mother and child increases for later wake times.

The limits of agreement in total sleep time between mother and child in the day shift group when compared to the night shift group show little difference. The average difference between mother and child in the day shift group was −54 min (mother sleeps less than child), with agreement ranging between −2 h 51 and 1 h 03. On average, the total sleep time for a mother and child pair was between 5:00 and 8:00 h. In the night shift group, the average difference was 29 min (mother sleeps less than her child), with limits of agreement between −5 h 20 and 4 h 22. On average, the total sleep time for a mother and child pair was between 5:00 and 9:00, and the difference between mother and child increased simultaneously with the increase in total sleep time.

Sleep efficiency (SE) values suggested little difference between mother and child in the day shift group when compared to the night shift group. The average difference between mother and child in the day shift group was −0.57% (mother being less efficient than child), with agreement ranging between −11.24% and 10.10%. On average, the SE of a mother and child pair was between 75% and 90%. In the night shift group, the average difference was −3.85%, with limits of agreement between −29.34% and 21.65%. On average, sleep efficiency was between 75% and 90%. However, the difference between mother and child in this parameter changes, as, for lower values, the mother’s sleep efficiency is lower than that of the child, while for higher values, the mother has higher values than the child.

There is also little difference between day shift and night shift workers regarding the limits of agreement in the wake after sleep onset (waso) parameter between mother and child. The mean difference between mother and child in the day shift group was around −5.83 min (mother fragments sleep less than child), with agreement ranging between −88.04 and 76.37 min. On average, the waso of a mother–child pair was between 40 and 110 min. In the night shift group, the mean difference was 34.67 min, with agreement ranging between −213.93 and 283.67 min. On average, the waso of a mother–child pair was between 70 and 190 min, with only a single pair showing a very high waso.

There is little difference between day shift and night shift workers regarding the limits of agreement in the WEP. The average difference between mother and child in the day shift group was 0.80 awakenings (mother wakes up more during sleep than child), with agreement ranging between −19.93 and 21.52 awakenings. On average, the wep of a mother–child pair was between 13 and 26 awakenings. In the night shift group, the average difference was −5.61 awakenings, with limits of agreement between −29.82 and 18.60 awakenings. On average, the wep of a mother–child pair was between 13 and 31 awakenings.

Table 2 shows all the parameters analyzed by actigraphy.

## 3. Discussion

This study analyzed the impact of maternal work schedules—night or day—on the activity and sleep parameters of children and workers. This is an unprecedented study that investigated the topic from an ecological perspective, without any changes to school, work, or family routines. The results suggest that in addition to the negative effects on the quality of family relationships [33] (Perry-Jenkins et al., 2007), maternal shift work, especially night shift work, can negatively impact the activity and sleep parameters of both mothers and children.

Our results indicate that participants in the night shift group sleep later, with a sleep onset time in the group between 11:00 and 8:00, and an average difference of 2 h 17 between mother and child, which means that if children start sleeping at 11:00, mothers start, on average, after 2:00, and can reach 8:00 on workdays, which imposes a routine of irregular sleep/activity times on these workers. Children of night workers also likely go to bed later than day shift children, whose sleep onset times ranged from 11:00 to midnight. Although the start time in both groups is similar (11:00), it is possible that night shift workers delay the start time for much longer than day shift workers (between 11:00 and 8:00).

These results suggest a more closely aligned bedtime routine among day shift workers, a fact that is corroborated by the values of the 5 h least active period (L5), which in the day shift group presented a difference of 51 min in later bedtime for mother than for child, while in the night shift group, the difference was 1 h 47 (later bedtime for mother than for child), reflecting the mother’s habit of sleeping more during the day, while the children have the habit of sleeping at night. For day shift mothers, the L5 values also suggest that their sleep is more restorative/consolidated than that of their children, which seems to be the opposite for night shift mothers, as they are much more active during the 5 h of least activity due to their night work routine. The same occurs with the wake time parameter, where work-imposed schedules mean that in the day shift group, mothers wake up earlier than their children, while the opposite occurs in the night shift group.

It has been widely demonstrated that shift work leads to desynchronization between biological and social rhythms and increases the risk of developing numerous diseases [32,34] (Costa, 2010; Silva-Costa et al., 2023). A large portion of studies are carried out with nursing professionals [35] (Rosa et al., 2019), a professional category that is affected by irregular working hours that result in harm to health. These include changes in sleep routines, the harmful effects of which can apparently extend to children, considering that poor sleep is increasingly common among children and adolescents and, as a biopsychosocial factor, is influenced by cultural agents, beliefs, and family values, in addition to biological mechanisms [36] (Liu et al., 2022).

The last century has been notable in terms of the decline in sleep duration among adults, with repercussions for adolescents, whose reduction in sleep duration has averaged 1 h per night, leading to day shift sleepiness and emotional irritability [37] (Matriccioni et al., 2012), among other symptoms. Despite the individual characteristics that impact sleep, the influence of parents and the family context are decisive and just as important [38] (Giannotti et al., 2009), making clear the importance of the family environment for the development of good sleep habits from childhood, which does not seem to occur with the participants in this study.

The total sleep time of both groups reflects the same pattern as the other parameters in general, with little difference between the day shift group and the night shift group. However, it is possible to verify that mothers in both groups sleep less than their children (5 h on average for both groups of mothers) and that, therefore, maternal sleep duration is below the recommendation (≤6 h) [39,40] (Hirshkowitz et al., 2015; Watson et al., 2015). Insufficient sleep duration is a global public health problem associated with the lifestyle of a 24/7 society, in which there is a need for workforce availability throughout the 24 h of the day to perform uninterrupted tasks, such as hospital care, policing, and others [41] (Wells and Vaughn, 2012). The reduction in duration has been observed in both adults and children/adolescents [42] (Chattu et al., 2018), although in this study, only mothers had a reduction; this fact is worrying, as sleeping less than necessary causes numerous adverse health effects and a high risk of morbidity and mortality [43] (Kim et al., 2013), in addition to representing a substantial problem for the economic sector.

In both cases, mothers have lower sleep efficiency (SE) compared to their children. However, among mothers who work night shifts, this difference is even greater, possibly due to the effect of night work on sleep, which can result in more superficial sleep during the day due to the social and environmental context in which sleep during the day is inserted [44] (Zhang et al., 2023) (during the day, mothers need to wake up more frequently to attend to their children and other household needs, a situation that is less frequent for mothers who sleep at night). Thus, we observed less agreement between night-sleeping mothers and their children in this parameter, which suggests a decrease in the effect of the mother’s inverted sleep pattern on the child itself, which, in this context, we consider to be positive.

The day shift group fragments sleep less than the night shift group, and mothers in this group have less fragmentation than their children. In day shift mothers who consolidate their sleep at night, the number of awakenings is lower compared to their children, who exhibit more restless sleep, probably due to their age (child/adolescent), for example due to the use of technologies, delayed sleep phase, among others [45] (Owens and Weiss, 2017). Among night shift workers, waking up is probably more frequent to take care of children, husbands, and other household activities during the day, since they sleep during the day, which reflects the longer duration of waking.

The results of the M10 parameter demonstrated that the participants and their children in the day shift group are more active during the day, with a smaller difference in this parameter between mothers and children (53 min average difference between the start of mothers and children, with mothers starting earlier), the opposite of night shift participants. Additionally, day shift children were more active than their mothers, which would be expected, since they are in the childhood/adolescence phase, with a tendency to practice more sports, play outdoors, and go to school, among other activities during the day. The same result was not observed in the night shift group, whose difference is greater (1 h 02, with mothers starting later) and increases for later M10 starts, which reflects the activity carried out at night by mothers and suggests that children may also remain active during the night for longer than children of day shift mothers, although the difference is not extreme between the groups. It can be inferred from the M10 values that participants in the day shift group have a more active lifestyle than those in the night shift group.

The interdaily stability (IS) quantifies the consistency of activity and rest rhythms across different days. A high IS indicates a regular sleep pattern, whereas a low IS suggests fluctuations and irregularities in sleep–wake cycles throughout the days. In this study, the values indicate less stability for the night shift group, that is, a less constant rhythm over the 24 h and different from the day shift group, but the difference is small. It is recognized that night workers may have difficulty synchronizing with light and dark due to their work schedules [46] (Boivin et al., 2022) and greater circadian misalignment.

Intraday variability (IV) indicated greater fragmentation of the rhythm in the night shift group. The greater variability also indicated a lack of agreement in this parameter between mothers and children. Among the night shift workers, only one pair (mother and child) indicated an unfavorable IV for the child (negative difference). For the other participants, the difference is positive, indicating higher values for mothers in relation to their children, which demonstrates more fragmented sleep among night mothers.

There was a considerable difference in the relative amplitude (RA) of the rhythm of the day shift and night shift groups. In the night shift group, there was lower RA among mothers when compared to their children, possibly given the participants’ work schedule, indicating that the rhythm is more “solid” among children and showing a difference between the activity and rest phases, as expected [47] (Fekedulegn et al., 2020). Meanwhile, there is a smaller difference between activity and rest for night shift workers in relation to the day workers. Night working reduces the amplitude of circadian rhythms [48] (Sack et al., 2007), and our results supported this statement, as among day shift participants, the difference observed was slight, with higher values than those of night shift participants. Among the night workers, all had lower RAs than their children (all points were in the negative ranges), which indicated less solidity of the sleep–wake cycle as a consequence of night work.

Greater instability and fragmentation in the activity and rest rhythm were expected among participants, especially those working night shifts, which was confirmed, characterizing circadian misalignment. What is surprising is that the differences were not significant between the groups, with participants in the day shift group also showing signs of misalignment, which compromises professional performance, the quality of rest, and family relationships, as described in the study by Coles et al. The authors highlight that poor sleep in children is negatively associated with the quality of family relationships, both between the couple and between parents and children [49] (Coles et al., 2022). The information obtained by the IV and IS parameters stands out, reflecting the rhythm and synchronization of the participants, with high IV values suggesting participants with fragmentation and possibly worse sleep, and lower IS values indicating less synchronization with the environmental light–dark period. Finally, the findings indicate that the variety of negative effects of shift work involve different domains that are interdependent (social, organizational, health) and demand a holistic approach. This study suggests the presence of family temporal desynchronization, with maternal work schedules disrupting the family context—particularly the activity and sleep parameters of their children.

## 4. Materials and Methods

An observational study was carried out in a large hospital in the city of São Paulo, Brazil. The final sample was defined as can be seen in the study’s explanatory model in Figure 1.

### 4.1. Participants

**Figure 3:**
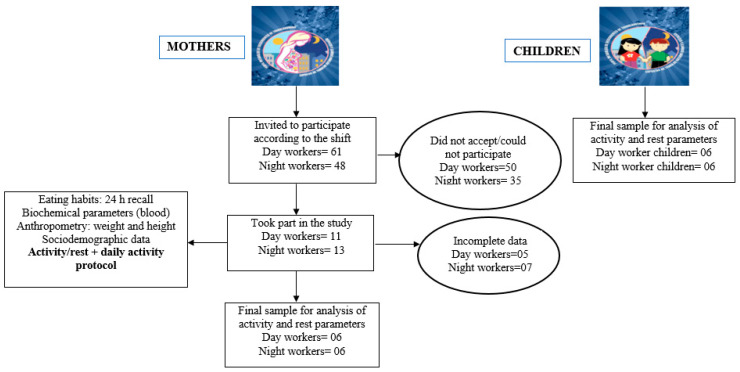
Study design.

Forty-eight (48) night workers and sixty-one (61) day workers and their children were invited to participate in the study. After applying the inclusion and exclusion criteria, our sample included 11 day workers and 13 night workers. For this specific analysis of activity rest data, we included only participants who correctly used the actigraph. Thus, the final study population (Figure 3) consisted of 12 nursing professionals: 6 night shift workers and 6 day shift workers (≥40 years old, 48 ± 5.89 years for the day shift and ≥40 years old, 50 ± 7.23 years). The children of these workers, with similar ages and gender (boys), were also included (*n* = 12), with 6 children of day shift workers and 6 children of night shift workers (day workers aged 10 ± 4.83, and in the night shift group, 10 ± 4.8 years) for the analysis of variables.

Participants worked a 12/36 h work schedule (7:00 to 19:00 for day shift workers and 19:00 to 7:00 for night shift workers).

The study was approved by the ethics committee of the School of Public Health of University of São Paulo (process n^o^. 1.583.205) and the Ethics Committee of the hospital where the study was carried out. Informed Consent Forms were obtained prior to the start of the study.

Workers (mothers) who had worked for at least 5 years in their current work shift (day or night) participated in the study. Night workers were required to have worked the night shift before and during pregnancy. Participants with acute or chronic illnesses, who had recently undergone surgery (12 months), who were taking medication, with a second job, or who had sleep problems were excluded. By including the mother, the children were automatically included, except for hospitalized children or carriers of acute or chronic diseases.

During the data collection period, the participants adhered to their regular schedules, specifically a 12/36 h work schedule (7:00 to 19:00 for day shifts; 19:00 to 7:00 for night shifts). Participants did not have any full days off between shifts throughout the data collection (i.e., two consecutive days or nights without work) during the 10-day study period. Due to the nature of their work schedules, not all participants began data collection on the same day of the week, rendering a uniform start date unfeasible. However, all participants commenced data collection on a workday, albeit on different weekdays, in accordance with their individual shifts. None of the participants initiated data collection during the weekend. All workers were exclusively assigned to either day or night shifts. There were no participants working dual jobs or alternating between day and night shifts.

### 4.2. Study Protocol

Actigraphy was used to monitor participants’ activity–rest cycles (ActTrust, Condor Instruments^®^ actigraph, City of São Paulo, State of São Paulo, country Brazil). In addition, a structured questionnaire was administered to collect sociodemographic information.

All data collection procedures were conducted at the participants’ workplace. During the initial meeting, potential participants were invited to take part in the study. The study objectives and procedures were explained, including instructions on the proper use of the actigraph, and the sociodemographic questionnaire was completed. This first session also included a general interview to collect baseline data.

The second meeting took place during the participant’s subsequent work shift and included the presence of their child. At this point, weight and height measurements were taken for both mother and child, and any remaining questions regarding the activity diary were addressed. Children also received training on how to properly wear the actigraph and complete the activity diary.

The following meetings were dedicated to monitoring adherence to the study protocol, verifying proper use of the devices, and clarifying any additional questions. Each participant and child wore the actigraph continuously and completed the activity diary over a 10-day period order to include at least one weekend in our study. All participants were instructed to maintain their usual routines throughout the study period to minimize behavioral changes due to study participation.

### 4.3. Anthropometric Data

To collect data regarding anthropometric measurements, participants were weighed on a calibrated analog scale with a capacity of 150 kg, with participants being barefoot and wearing light clothing. Height was measured using a wall-mounted stadiometer without baseboards. The data were used to calculate the Body Mass Index (BMI), applying the reference values established by the World Health Organization [50] (WHO, 2000), according to the following categorization: BMI < 18.5 underweight; eutrophy ≥ 18.5 and ≤24.9; overweight ≥ 25.0 and ≤29.9; and obesity ≥ 30 kg/m^2^. The same procedure was carried out with the children using the reference standards for children [51] (WHO, 2007).

### 4.4. Activity and Sleep Data

Actigraphy data were collected in 1 min epochs and analyzed using Actstudio software. The analysis of the activity and rest rhythm were used to characterize the parameters: the period of 5 less active hours (L5), calculated from the average of the period of 5 consecutive hours with less movement, considering the standard average of movement over 24 h; the period of 10 most active hours (M10), which is calculated as the average activity in the 10 consecutive hours of greatest activity; the interdaily stability (IS), which quantified the constancy of rhythm between 24 h periods; the intraday variability (IV), which quantified the fragmentation of the rhythm and the alternation of transitions between rest and activity within 24 h; and relative amplitude (RA), which measures the difference between M10 and L5. Additionally, parametric data were analyzed: bedtime and wake time, total sleep time, duration, latency, and sleep efficiency, as well as number of awakenings.

The activity diary was used to complement the information generated by the actigraph. Thus, they were completed daily, over the course of 24 h, to provide data on sleep and wake times over the 10 days of study, including naps. The bedtime and wake time reported in the diary helped determine the interval for daily analysis of objective actigraphic sleep parameters.

### 4.5. Data Analysis

Sleep and motor activity parameters obtained by actigraphy were analyzed using Pearson’s correlation coefficient and the Bland and Altman technique [52] (Bland and Altman, 1986). This technique considers A and B according to measurements provided by the actigraphy of the mother and child, respectively. In this case, different measures of mothers and children were used to assess the degree of agreement among them.

For each pair, the average [(A + B)/2], the difference (A–B), the average difference, and the standard deviation of the differences were calculated. The mean difference corresponds to the estimated bias, that is, the systematic difference between mothers and children: zero average differences reflect perfect agreement, positive average differences indicate an overestimation of the mother’s measurement, and negative average differences indicate an underestimation.

The standard deviation measures random fluctuations around this average, and the 95% limits of agreement (average difference plus or minus 1.96 standard deviations) provide a maximum distance estimate between mother and child, for most pairs.

## 5. Conclusions

Our results should be interpreted with caution due to the limited sample size, which precludes drawing definitive conclusions about the magnitude of the observed effects. Nonetheless, the findings provide preliminary evidence suggesting a potential influence of maternal work schedules on children’s sleep patterns, warranting further rigorous investigation. The data suggest that maternal night shift work can have a negative impact on the activity and sleep parameters of both mothers and children compared with day shift. Strategies to mitigate sleep deprivation should be implemented, alongside initiatives to promote sleep hygiene education. Additionally, future research should explore family-centered approaches to addressing this issue to better understand and intervene in the interdependent sleep dynamics of mothers and children.

## 6. Limitations

The number of participants was reduced due to the complexity of the study protocol, which was implemented without altering participants’ daily routines. For the same reason, other hospitals were not included in the study. Additionally, matching mothers and children by age and gender posed a significant challenge. Although the attrition rate represents a limitation, efforts were made to minimize its impact by selecting participants with similar characteristics (age and gender).

## Figures and Tables

**Table 1:** Sociodemographic characterization of participants and their children, in absolute and relative frequency, according to work shift (*n* = 24).

Variables	Day Workers and Children (*n* = 12)				Night Workers and Children (*n* = 12)			
	Mothers		Children		Mothers		Children	
	*n* %	Mean (sd) [Range]	*n* %	Mean (sd) [Range]	*n* %	Mean (sd) [Range]	*n* %	Mean (sd) [Range]
Age								
Years	6 (100)	47.67 (2.42) [6]	6 (100)	7.83 (1.47) [4]	6 (100)	49.6 (4.97) [8]	6 (100)	9.17 (0.75) [2]
Level of education								
Elementary and middle school	0 0	n.a.	6 100	n.a.	0 0	n.a.	6 100	n.a.
High school	6 100	n.a	0 0	n.a.	5 83.3	n.a.	0 0	n.a.
Undergraduate degree	0 0	n.a.	0 0	n.a.	1 16.7	n.a.	0 0	n.a.
Nutritional Status								
Eutrophy	2 33.3	n.a.	5 83.3	n.a.	2 33.3	n.a.	4 66.7	n.a.
Overweight	2 33.3	n.a.	1 16.7	n.a.	1 16.7	n.a.	2 33.3	n.a.
Obesity	2 33.3	n.a.	0 0	n.a.	3 50.0	n.a.	0 0	n.a.
Net family income								
≤BRL 4000.00	6 100	n.a.	n.a.	n.a.	5 83.3	n.a.	n.a.	n.a.
≥BRL 4001.00	0 0	n.a.	n.a.	n.a.	1 16.7	n.a.	n.a.	n.a.
Professional category								
Nurse	0 0	n.a.	n.a.	n.a.	1 16.7	n.a.	n.a.	n.a.
Nursing assistant/technician	6 100	n.a.	n.a.	n.a.	5 83.3	n.a.	n.a.	n.a.
Current marital status								
Single	2 33.3	n.a.	n.a.	n.a.	0 0	n.a.	n.a.	n.a.
Married/lives with partner	4 66.7	n.a.	n.a.	n.a.	4 66.7	n.a.	n.a.	n.a.
Separated/divorced/widowed	0 0	n.a.	n.a.	n.a.	2 33.3	n.a.	n.a.	n.a.
Total time of sleep (h)	n.a.	6 h 20 (1 h 02) [5 h 43–8 h 04]	n.a.	7 h 13 (01 h 25) [5 h 9–9 h 06]	n.a.	7 h 06 (01 h 54) [5 h 8–10 h 12]	n.a.	7 h 35 (01 h 42) [5 h 19–10 h 20]
Diurnal motor activity, M10 (counts/min)	n.a.	4920.7 (1133.5) [3807.2–6773.5]	n.a.	5734.4 (1539.9) [4474.2–8671.0]	n.a.	4138.1 (727.6) [3645.5–5124.7]	n.a.	4506.4 (2489.5) [1630.4–8629.4]
Nocturnal motor activity, L5 (counts/min)	n.a.	332.21 (105.24) [252.2–493.6]	n.a.	390.78 (271.89) [150.9–741.3]	n.a.	1250.74 (290.15) [911.3–1767.1]	n.a.	562.36 (320.90) [204.1–1152.1]

n.a.: not applicable.

**Table 2:** Activity parameters of mothers and children, assessed by actigraphy, according to the work shift of the mother.

Actigraphy	Parameter	Day Workers (Mothers and Children)			Night Workers (Mothers and Children)		
		Agreement Range	Average Difference in Group	Average	Agreement Range	Average Difference	Average
	L5 (counts/min)	−454.96 and 337.81	−58.57	500	−107.8 and 1485.57	688.39	1200
	L5 onset (h)	1 h 42 and 3 h 24	51 min	11:00–12:00 *	−4 h 26 and 8 h 18	1 h 47 *	11:00 and 7:00
	M10 (counts/min)	−2347.86 and 1257.81	545.03	4500 and 7500 **	−5494.61 and −4757.97	−368.32	2500 and 6500 **
	M 10 onset (h)	−4 h 22 and 6 h 17	53 min	7:00 and 2:30 *	−8 h 47 and 10 h 51	1 h 2	7:00 and 2:00 *
	IS	−0.30 and 0.28	−0.01	0.5 and 0.65 **	−0.20 and 0.27	0.03	0.20 and 0.40 **
	IV	−0.19 and 0.2	0.04	0.6 and 0.9 *	−0.36 and 0.67	0.15	0.5 and 1.1
	Relative amplitude (RA)	−0.13 and 0.16	0.01	0.7 and 0.9 **	−0.41 and −0.04	−0.22	0.5 and 0.7 **
	Bedtime (h)	−2 h 14 and 2 h 17	8 min	11:00–12:00 *	−11 h 40 and 15 h 37	2 h 17	11:00 and 8:00 *
	Wake time (h)	−4 h 37 and 3 h 18	−40 min	7:00 and 10:00 *	−9 h 45 and 11 h 53	1 h 4	7:00 and 3:00 *
	Total time of sleep (h)	−2 h 51 and 1 h 3	−54 min	5:00 and 8:00 *	−5 h 20 and 4 h 22	29 min	5:00 and 9:00 *
	Sleep efficiency (%)	−11.24 and 10.10	−0.57	75 and 90 **	−29.34 and 21.65	−3.85	75 and 90 **
	WASO (min)	−88.04 and 76.37	−5.83	40 and 110 **	−213.93 and 283.67	34.67	70 and 190
	WEP	−19.93 and 21.52	0.80	13 and 26 **	−29.82 and 18.60	−5.61	13 and 31 **

* Average start time of the parameter in the mother and child pair. ** Average parameter value for mother and child pair.

## Data Availability

The original contributions presented in this study are included in the article. Further inquiries can be directed to the corresponding author(s).
